# “Just a second, mommy’s here”: the impact of mothers’ smartphone use on children’s affect regulation and the quality of mother–child interactions

**DOI:** 10.3389/fpsyg.2025.1596219

**Published:** 2025-07-09

**Authors:** Aleksandra Mikić, Sarah Bergmann, Georgina Perejoan Martí, Annette M. Klein

**Affiliations:** ^1^International Psychoanalytic University Berlin, Berlin, Germany; ^2^Department of Developmental Psychology, University of Potsdam, Potsdam, Germany; ^3^Department of Child and Adolescent Psychiatry, Psychotherapy and Psychosomatics, University of Leipzig, Leipzig, Germany

**Keywords:** mother–infant interaction, infant affect regulation, emotional availability, maternal responsiveness, smartphone use, technoference, media multitasking

## Abstract

**Introduction:**

The ubiquity of smartphone devices in our everyday lives has been widely recognized as a potential challenge to the quality of parent–child interactions. The aim of this study was to experimentally examine the effects of mothers’ smartphone use on their children’s affect regulation and on the quality of mother–child interactions, indicated by emotional availability of the dyad and maternal responsiveness. Additionally, we investigated the associations between mothers’ behaviors to maintain contact with their children during smartphone use and their children’s affect regulation.

**Methods:**

The experiment consisted of two counterbalanced phases: the free play phase and the interruption phase, in which mothers were replying to standardized text messages in the presence of their children. The sample comprised 52 mothers and their children aged 5 to 6 months (24 female).

**Results:**

Infants expressed less positive affect in the interruption phase than in the free play phase, as well as more negative affect in the interruption phase when the free play phase preceded the interruption phase. In addition, the mothers showed less sensitivity and responded to less infants’ signals and in a slower way in the interruption phase than in the free play phase. Moreover, mothers showed less optimal structuring, and children showed less well involvement of their mothers in the interruption phase than in the free play phase. Lastly, more children’s negative affect was associated with a shorter duration of mothers’ smartphone use and more active mothers’ behaviors to maintain contact with their children during the smartphone use.

**Discussion:**

These results suggest that, although mothers adapt smartphone use based on their children’s affective response, mothers’ repeated smartphone use negatively impacts their children’s affect regulation and the quality of the mother–child interactions, with potentially negative consequences for the children’s social–emotional development.

## 1 Introduction

Digital technology is increasingly shaping our attentional and communication habits, with portable digital devices (PDD) such as smartphones being attributed a particularly strong influence due to their ubiquity in everyday life (Fitz et al., 2019; Oulasvirta et al., 2011). The widespread habit of media multitasking (e.g., media use when involved in non-media activities; van der Schuur et al., 2015) implies a frequent interference of device use with social interactions—a phenomenon termed technoference (McDaniel and Coyne, 2016). In parents of young children, sharing attention between caregiving and PDD use might be a practical solution when managing numerous everyday tasks and needs. However, when multitasking, perception of children’s communicative signals might be compromised.

In infancy, children are largely dependent on their primary caregivers, usually their mothers, to regulate their affective states (e.g., Sameroff, 2014). Parental sensitivity, i.e., the ability to accurately perceive and correctly understand children’s signals and to respond to them promptly and appropriately, is seen as essential for an effective interactive regulation (Ainsworth et al., 1978; Gianino and Tronick, 1988; Lyons-Ruth, 1999). In contrast, parental unresponsiveness might lead to a dysregulation of infants’ affect, expressed by, e.g., crying. This is illustrated by studies using the still-face paradigm, in which parents are instructed to maintain a still face and remain unresponsive to their children’s signals after a period of free play, resulting in distress in the children (Tronick et al., 1978). Over time, regulating behaviors of caregivers are thought to be internalized by the children and to shape their ability to self-regulate their affect (Cassidy, 1994; Fonagy et al., 2005; Sroufe, 1996), which is of great importance for their future social–emotional development (e.g., Thompson, 1994). As such, frequent emotional dysregulation occurring during early mother–infant interactions is a risk factor for psychopathology later in life (Keenan, 2000; Cole et al., 1994).

The potential of parents’ PDD use in the presence of their children to compromise both perception of the children’s cues and the quantity and quality of reactions to them, with potentially negative consequences for children’s affect regulation has been increasingly recognized in both media and scientific inquiries. A further question arises regarding the influence on aspects of the interaction quality beyond parental sensitivity and responsiveness, such as the emotional availability of the parent–child dyad. Emotional availability (EA), defined as the ability of a dyad to share a healthy emotional connection, is a theoretically and empirically well-established indicator of the quality of the early interactions (Biringen et al., 2014; Saunders et al., 2015). It is grounded in the attachment theory but apart from exploring adults’ sensitivity, it encompasses various other components of adults’ and children’s behavior important for the children’s social–emotional development, such as adults’ structuring of interactions with their children (Saunders et al., 2017). An important issue to consider is whether parental PDD use inevitably impairs parents’ sensitivity and other aspects of the emotional availability of the dyad or if adjusting the usage to the requirements of the situation, such as by reducing the usage time or by maintaining contact with the children during the usage period, could be sufficient for sustaining a high quality of interaction.

Parents could use different strategies to maintain contact with their children while using a PDD device. We assume that parental device use often involves frequent switching of attention between the PDD and the children (sequential multitasking, i.e., task-switching) or even a simultaneous interaction with the children (concurrent multitasking; Salvucci et al., 2009; herein referred to as multitasking). It is important to investigate whether parents employ such strategies of maintaining contact with their children, and how it impacts children’s affect regulation.

A growing number of studies explore the effects of parental technoference on children’s affect regulation and the quality of parent–child interactions. In the following overview of previous research, we focus on studies with children aged 0–3. Two recent studies modified the still-face paradigm by instructing parents to exhibit a still face while looking at a PDD (without using it), and were able to largely replicate the typical still-face effect (for a review see Mesman et al., 2009): Infants and toddlers expressed more negative and less positive affect during the still-face phase compared to the free play phase, with evidence for a carry-over effect in the reunion phase (Myruski et al., 2018; Stockdale et al., 2020). These findings indicate that prolonged parental immersion in a PDD can be dysregulating for infants. Nevertheless, if parents actively use a PDD it might not have an equally disruptive effect on the parent–child interaction. In one further experimental study, mothers were randomly assigned to either answer messages via mobile phone or to reply to an experimenter’s questions in person or to play undisrupted with their 10-month-old infants. All conditions were preceded and followed by a free play (Rozenblatt-Perkal et al., 2022). In both disruption conditions, infants had a higher heart rate (as a measure of physiological arousal) and expressed more negative affect in the texting/conversation phase compared to the preceding free play phase. The effect of the disruption on infants’ negative affect was more pronounced when mothers used a mobile phone than when talking to the experimenter. Moreover, in the mobile phone disruption condition no carry-over effect was found: The expressed infants’ negative affect in the subsequent free play phase was not significantly different compared to the preceding free play phase, suggesting no sustained impact of the interruption on the children’s affect regulation. In addition, in one experimental study with toddlers, children expressed more negative affect during the periods when their parents used a smartphone in comparison to the periods when their parents demonstrated an action to them (Konrad et al., 2021a). Overall, these experimental findings suggest that short-term technoference negatively influences infants’ affect during the episode but does not result in persistent dysregulation afterward. Nevertheless, some other findings indicate positive associations between parental habitual PDD use and everyday difficulties in infant affect regulation. Namely, in two cross-sectional studies based on questionnaire data and measuring infant temperament, mothers who reported more everyday technology use during feeding and care interactions with their infants reported more child negative affectivity (Alvarez Gutierrez and Ventura, 2021; Davis et al., 2022), and a lower orienting/regulatory capacity in infants aged 22.4 weeks, but not in younger infants (Davis et al., 2022). Moreover, results of a recent study showed that higher levels of parents’ immersion during smartphone use are associated with a more difficult temperament in infants (Wade-Bohleber et al., 2024). However, the causal relationships between these aspects of parental technoference and infant temperament are yet to be understood.

To date, only a few studies have investigated the quality of parent–child interactions—especially parental sensitivity and responsiveness—during parents’ PDD use in the early years, resulting in inconsistent findings (for reviews, see Braune-Krickau et al., 2021; Mikić and Klein, 2022). In a study based on a naturalistic observation of parents and their children aged 0 to 3 years at playgrounds, a longer duration of mothers’ smartphone use was associated with lower levels of sensitivity towards their children whereas the frequency of use was not associated with maternal sensitivity (Wolfers et al., 2020). Utilizing a structured observation of mothers interacting with their 3-7-month-old infants, Tharner et al. (2022) found lower mothers’ sensitivity during free play to be associated with longer everyday smartphone use while being alone with their infants, but not with the frequency of picking up the smartphone (as measured by a passive sensing app). In an experimental study, mothers were instructed to breastfeed their infants aged 2 to 6 months and to either use their smartphone as they would at home (without further instructions) or not to use it (Inoue et al., 2022). No significant difference was found in the quality of mother–child interaction in the smartphone use condition compared to the control condition. However, it is important to note that the very small sample sizes of the two aforementioned studies limit the generalizability of the findings. In another experimental study, mothers watched a TV show on a tablet while breastfeeding their infants aged 6.2 to 32 weeks. Results showed that the mothers were not less sensitive to infants’ cues during the digital media use condition compared to the control condition (in which mothers were listening to ambient-level classical music), although there was a trend in this direction (Ventura et al., 2019). There was no difference in mothers’ responsiveness to infants’ distress between the two conditions. However, the sample size was small and the type of PDD use too specific to conclude that PDD use has no effect on maternal sensitivity and responsiveness. In particular, the level of one’s absorption in the content of a PDD may be lower when watching a TV show than during an interactive and dialogical device use (Gergen, 2002). Another experimental study involving parents and their children aged 12–36 months found that the quality of parent–child interactions (as measured by parental sensitivity, dyadic reciprocity and dyadic negative states) significantly decreased when parents were distracted by completing a questionnaire on either a tablet or a printed form and were significantly lower compared to a control group, in which parent–child interactions were uninterrupted (Chamam et al., 2024). However, the wide age range may have masked age-specific effects of parental distraction. Further experimental studies exploring parent–child interactions in toddlerhood point to a decrease in parental responsiveness when using a PDD in the presence of their children. Two studies involving toddlers found reduced maternal responsiveness to their children when mothers were engaged with smartphone use, compared to when they played with their children without interruption (Konrad et al., 2021b; Lederer et al., 2022). However, there is still a research gap regarding parents’ responsiveness to their children during PDD use (especially interactive use) in the first year of life.

There are reasons to assume that parental behaviors aimed at maintaining contact with their children—such as touching or talking to them—may reduce the disruptive effects of parental PDD use. Previous studies using modified still-face experiments found that touch ameliorates the distress children display when confronted with their mothers’ still face (Stack and LePage, 1996; Stack and Muir, 1992) and that touch helps infants to recover more effectively during the reunion phase (Feldman et al., 2010). However, not all parental behaviors seem to be equally effective. For example, Stack and Muir (1992) showed that active rather than static touch positively modulates the infants’ affect during the still-face experiment. Likewise, it seems that not every communication modality is equally effective to maintain contact with infants. Findings of another modified still-face experiment showed that there was no difference in infants’ reactions between a condition where mothers talked to their children while keeping a still face and the standard still-face condition without talking (Gusella et al., 1988). Perhaps this misalignment between facial and verbal communication was just as disturbing to children as their mothers’ complete lack of response. Even if contact is maintained while parents are using a PDD, the interaction could be of lower quality and potentially dysregulating for the infants compared to an undisrupted interaction. Such interactions might entail a lower degree of interpersonal contingency (i.e., predictability of behavior over time; Beebe et al., 2010). For example, during instances of multitasking involving a PDD, parental gaze and facial expressions are often not or only partially aligned and coordinated with those of the child. In instances of task switching, the flow of interaction could be repeatedly interrupted. Therefore, the result might be the lack of coherence and continuity of the infants’ experience, possibly leading to confusion and frustration in the infants.

To date few studies have investigated children’s affective responses to caregivers’ PDD use and research exploring impacts of parental technoference on the quality of parent–child interactions in the first year of life is still scarce. Moreover, very little research was conducted in a controlled setting, and previous experimental studies focused largely on the effects of temporary interruptions (PDD use lasting from 30 s to 3 min). Also, in some of these studies parents were explicitly instructed not to react to their children’s bids. However, in everyday situations, parents may coordinate their PDD use with ongoing interactions with their children. While this may lead to repeated interruptions, it could also offer opportunities to maintain a reasonably high quality of interaction. Finally, to our knowledge, no studies so far explored associations between parental behaviors to maintain contact with their children while using a PDD and the children’s affective response.

With the aim of addressing these research gaps, we designed an experiment consisting of two phases: the free play phase and the interruption phase, in which mothers received standardized text messages on a smartphone and were instructed to respond to them during the interaction with their children. We sought to simulate a typical everyday texting situation by designing the experimental instructions accordingly and allowing sufficient time for mothers to coordinate smartphone use with interactions with their children.

We decided to focus on infants aged 5 to 6 months, given their substantial reliance on parental co-regulation (e.g., Calkins, 2004). We chose to examine the effects of a form of smartphone use that involves content production, i.e., texting, given its high prevalence in everyday life and its greater cognitive demands compared to passive consumption of content (Cabañero et al., 2020). As there are still many gaps in understanding the impact of parents’ PDD use on the quality of parent–child interactions, we employed both a global coding system (EA of both members of the dyad) and an event-based coding system (mothers’ responsiveness to their children’s vocal communications) to comprehensively evaluate the quality of mother–child interactions. Lastly, to understand the ways mothers maintain contact with their children during PDD use, we developed a scale measuring these behaviors.

Based on the existing findings we hypothesized that: (1) infants will express less positive and more negative affect in the interruption phase than in the free play phase of the experiment; (2) maternal sensitivity, as an aspect of EA of the mother–infant dyad, will be lower in the interruption phase than in the free play phase; (3) mothers will respond less often and slower to infants’ vocal signals in the interruption phase than in the free play phase.

In addition, we investigated the following exploratory research questions: (1) Do further aspects of EA differ between the interruption and the free play phase of the experiment? (2) Are (a) the duration of mothers’ smartphone use during the interruption phase and (b) the amount of active and/or passive behaviors to maintain contact shown by mothers during the interruption phase associated with the positive and negative affect expressed by infants in the same phase of the experiment?

## 2 Materials and methods

### 2.1 Design

The experiment had a 2 (within-subjects) × 2 (between-subjects) design. It consisted of two phases (within-subjects) each lasting 8 min: the free play phase, in which mothers were asked to play with their children as they normally would and the interruption phase, in which mothers received standardized text messages on a smartphone and were instructed to respond to them during the interaction with their children. The order of the phases was counterbalanced across participants (i.e., between-subjects): For one half of the sample, the experiment started with the free play phase, for the other half with the interruption phase. Although no hypotheses were formulated regarding the order of experimental phases, this between-subjects factor was included in the analyses, as well as the interaction effect (phase × condition) to control for potential confounding effects. In the interruption phase, mothers received up to four messages via WhatsApp. The time the first message was sent to the participants was standardized, and subsequent messages were sent only after mothers replied to the previous one. Most of the mothers received all four messages (96.2%). 80.8% of mothers responded to all four messages, 15.4% responded to three, 1.9% responded to two and 1.9% responded to one message. For most cases of unanswered messages, the replies had been typed, but not sent, or the experimental phase ended before the participants had finished replying. Some mothers (3.8%) posed additional questions, asking for clarification, and those questions were replied to shortly.

### 2.2 Participants

Infants aged 5 months to 6 months and 15 days, who were born full term (≥ 37 gestational weeks), with a birth weight equal to or above the 10th BMI-percentile and who were typically developing were eligible to participate in the study. The inclusion criteria for mothers were ownership of a smartphone with an iOS or Android operating system and being on maternal leave (accessible to all families in Germany) or working less than 20 h per week. The participants were recruited through social media, midwives’ private practices, recommendations by other participating mothers, infant programs, family centers, and flyers. Data collection took place between July 2021 and August 2022.

Focusing on the first study hypothesis, sample size estimation using G*Power (version 3.1.9.4.; Faul et al., 2007) for repeated measures ANOVA was performed. Based on earlier findings we assumed a small to medium effect size (meta-analysis, Mesman et al., 2009; lowest effect size for an increase in negative affect between the free play and the still-face phase). An *a priori* test with an effect size *f* = 0.225, *α* = 0.05, power = 0.80, within-subjects factor phase = 2, between-subjects factor order = 2 and moderate correlations between repeated measures (*r* = 0.40) showed that *N* = 50 of mother–infant dyads are needed for analyzing differences between phases. We included two more dyads to increase the power of the analyses. A total of 67 dyads attended the experimental session, but *n* = 7 were excluded due to infants’ excessive crying (> 50% of time), *n* = 5 because the mothers did not follow the experimental procedure and *n* = 3 due to errors in the testing procedure. The final sample consisted of *N* = 52 infants and their mothers. All mothers were biological mothers. Further sample characteristics are presented in Table 1.

**Table 1:** Sociodemographic characteristics of the sample.

Variable	Condition 1 (*n* = 26)	Condition 2 (*n* = 26)	Total sample (*N* = 52)
Children’s characteristics			
Age (Months) *M* (*SD*)	5.86 (0.47)	5.70 (0.38)	5.78 (0.43)
Gender, Female, *n* (%)	12 (46.2)	12 (46.2)	24 (46.2)
Family structure			
Lives with both parents	26 (100)	25 (96.2)	51 (98.1)
Lives with mother only	0	1 (3.9)	1 (1.9)
Siblings *n* (%)			
No siblings	12 (46.2)	13 (50.0)	25 (48.1)
One	8 (30.8)	11 (42.3)	19 (36.5)
Two	6 (23.1)	2 (7.7)	8 (15.4)
Mothers’ characteristics			
Age (Years) *M* (*SD*)	33.15 (3.28)	33.42 (4.54)	33.29 (3.92)
Education *n* (%)			
Elementary/secondary school degree	0	2 (7.7)	2 (3.8)
Vocational Training	4 (15.4)	7 (26.9)	11 (21.1)
University Degree	22 (84.6)	17 (65.4)	39 (75.0)
Citizenship *n* (%)			
German	24 (92.3)	25 (96.2)	49 (94.2)
German and other	2 (7.7)	0	2 (3.8)
Other	0	1 (3.8)	1 (1.9)
Fathers’ characteristics^a^			
Age (Years) *M (SD)*	35.08 (4.69)	35.92 (6.76)	35.49 (5.75)
Education *n* (%)			
Elementary/secondary school degree	1 (3.8)	2 (8.0)	3 (5.9)
Vocational Training	8 (30.8)	9 (34.6)	17 (33.4)
University Degree	17 (65.3)	14 (53.9)	31 (60.7)
Citizenship *n* (%)			
German	21 (80.8)	20 (80.0)	41 (80.4)
German and other	1 (3.8)	1 (4.0)	2 (3.9)
Other	4 (15.4)	4 (16.0)	8 (15.7)
Household income *n* (%)			
1,092–1971 EUR	2 (7.7)	2 (7.7)	4 (7.7)
1972–2,833 EUR	3 (11.5)	2 (7.7)	5 (9.6)
2,834 EUR or more	21 (80.8)	22 (84.6)	43 (82.7)

a *n* = 51.

### 2.3 Procedure

The study was reviewed and approved by the Ethics Committee of the International Psychoanalytic University Berlin. Mothers who were interested in participating in the study received detailed information about the study in written and oral form. Participation in the study was voluntary. For mothers who provided informed consent, an appointment was scheduled, and they were instructed to complete online questionnaires. In addition, they were asked to install the ExperienceSampler application (Thai and Page-Gould, 2018) and answer short questionnaires when notified, during the period of 1 week before the appointment. Upon arrival at the testing room, mothers were provided with a general overview of the testing procedure. After getting comfortable, ECG electrodes were placed on both mothers’ and infants’ skin for measuring their heart rate during the experiment (the results of analyses involving experience sampling, questionnaire data, and physiological measures will be reported elsewhere). Mothers were then instructed to place their infants on a 120 × 120 cm mat and sit beside them. Next to the mothers was a box containing a standardized set of age-appropriate toys, a nursing pillow (as an optional back support for the infants), and a small table on which a smartphone was placed. The experimental smartphone ran either with iOS or Android operating system, depending on the operating system of the mothers’ own devices, and was used instead of participants’ personal smartphones to prevent interferences from private notifications. For this reason, mothers were also instructed to turn off their personal smartphones prior to the start of the experiment. Once the participants were ready, the baseline ECG-measurement and video recording (by three cameras capturing different angles) began. After 3 min, the baseline measurement concluded, and the experiment started. Mothers were asked to play with their children using the available set of toys. For the free play phase of the experiment, they were instructed to play with their children as they usually would. For the interruption phase, mothers were told that they would receive several text messages to which they should respond as quickly as possible, unless the situation required otherwise. Mothers were asked to imagine that a close friend was asking them for recommendations and to respond to the messages in written form as they would in everyday life. Depending on the assigned experimental condition, they were informed about the order of the phases. The transition from the first to the second phase was indicated by an auditory signal.

After the experiment had ended, mothers were informed about the objectives of the study in more detail and given the opportunity to ask questions. They received compensation of 40€ for their participation, along with a small gift and a certificate of participation for their children.

### 2.4 Measures

Mothers filled out questionnaires. Mothers’ and infants’ behaviors during the experiment were coded using the software Interact (except for the EA assessment; Mangold, 2018).

#### 2.4.1 Sociodemographic data

Sociodemographic data were collected through a questionnaire that included questions about the mothers’ and fathers’ age, education and employment status, family structure, and household income (see Table 1). The socioeconomic status was assessed based on items of the German Working Group for Epidemiology (Lampert et al., 2018).

#### 2.4.2 Infant affect

We used Repeated Still-Face Paradigm Infant Affect Codes (Bosquet Enlow et al., 2011) to code infants’ affect during the experiment. The scale consists of the following mutually exclusive codes: hard crying, crying, fussing/negative affect without vocalizations, neutral, positive, very positive, mixture of positive and negative affect, unclassifiable, unobservable/asleep, and autonomic indicator (yawning or sneezing when affect is not codable). Due to the length of the experiment, codes were scored at 10-s intervals for the entire duration of the experiment, based on the affect expressed during the majority of time within each interval. Two trained raters coded the data, and 23.1% of the videos were double coded for the reliability analysis. The agreement between the two raters was excellent, with ICC = 0.92 for positive affect and ICC = 0.99 for negative affect. The infants’ negative affect score was calculated separately for the two phases of the experiment by summing the proportions of time infants expressed hard crying, crying, or fussing/negative affect without vocalizations, with different weightings based on the intensity of affect (fussing + 2x crying + 3x hard crying; Bosquet Enlow et al., 2014). Analogously, the infants’ positive affect score was calculated by summing the weighted proportions of time infants expressed positive and very positive affect (positive + 2x very positive affect) in each of the experimental phases.

#### 2.4.3 EA of the dyad

We used the infant/early childhood version of the Emotional Availability Scales (EAS), 4th Edition (Biringen, 2008) to rate EA. The EAS consist of four scales that are used to assess the mothers’ contribution to the interaction, namely sensitivity, structuring, nonintrusiveness, and nonhostility, and two scales assessing the infants’ contribution, i.e., child responsiveness and child involvement. EA takes a relational perspective into account, i.e., none of the behaviors is assessed purely individually, but always in relation to the other member of the dyad. Based on the videotaped mother–infant interactions, the six EA dimensions were assessed in real time, and direct scores for each dimension (from 1 = non-optimal to 7 = optimal) were assigned separately for each phase of the experiment with higher scores indicating higher EA. The direct scores can be allocated to distinct categories indicating different constellations of interactive behaviors (Biringen, 2008).

One certified rater coded the data. Additionally, a second certified rater coded 25% of the videos to calculate interrater reliability, which ranged from ICC = 0.76 to ICC = 0.87, demonstrating good reliability. The construct validity of the EAS in relation to different caregivers, children, and dyadic constructs (for an overview, see Biringen et al., 2014), as well as its applicability in German samples (Bergmann et al., 2016), has been established.

#### 2.4.4 Maternal responsiveness

To code mothers’ responsiveness to their children’s signals, the Bornstein and Tamis-LeMonda scales (Bornstein et al., 1992; Bornstein and Tamis-LeMonda, 1997) were adapted to fit the examined age group and the experimental context. Specifically, we coded every vocal sound that the infants made (babbling, cooing, laughing, vocal games, screeching and sighing, crying, screaming), but excluded vegetative body sounds, grunting and sounds of making effort. Afterwards, we coded the mothers’ responses to these signals, defined as any observable change in their behavior that followed the children’s signals and appeared conceptually linked to the children’s behavior (such as gazing at children, vocalizing, talking, or touching children). The mothers’ and children’s behaviors were coded on a second-by-second basis for the entire duration of the experiment by two trained coders. Two scores were created separately for each phase of the experiment: (1) the frequency of responses—the percentage of the infants’ signals to which the mothers responded and (2) the latency of responses—the number of seconds from the moment the infants gave a signal until the mothers responded. The ICC analysis was conducted on 21.2% of the videos and showed excellent reliability for the frequency of responses (ICC = 0.97) and good reliability for the latency of responses (ICC = 0.88).

#### 2.4.5 Duration of smartphone use and maintaining contact with children

The communicative behaviors exhibited by mothers while using the smartphone, which were likely aimed at maintaining contact with their children, were coded using the Maintaining Contact Scale developed by the research team (see Supplementary Table 1). Similar to Goldsmith and Rogoff (1997), we measured maternal secondary attentional focus on children, while their primary focus was on the smartphone task. We focused on two types of behaviors: multitasking, defined as mothers’ simultaneous interaction with their children while using the smartphone, and task switching, defined as mothers’ interaction with their children between bouts of smartphone use. Based on the findings of Stack and Muir (1992), that active, but not passive maternal touch ameliorates the distress children display when confronted with their mothers’ still face, the coded behaviors were categorized as active or passive. Active behaviors comprise facial communication, interaction using one hand or toy, touching or stroking children, kissing children, moving children, taking children on the lap, in the arms or positioning them to sit between the mothers’ legs, rocking or swaying children, talking, singing, laughing or producing other communicative vocal sounds. Passive behaviors encompass holding one hand up or a toy over children, sustained touch, and keeping children on the lap, in the arms or between the legs. Maternal monitoring (i.e., gazing at children), and behaviors that were not clearly communicative and therefore could not be classified as behaviors to maintain contact (e.g., touching a toy that children cannot see) were treated as separate categories. All behaviors were coded on a second-by-second basis; active and passive behaviors could occur simultaneously. Two scores of behaviors to maintain contact—active and passive—were created, each encompassing multitasking and task switching. In addition, we calculated separate scores for multitasking and task switching: active multitasking, active task switching, passive multitasking, and passive task switching. The duration of smartphone use was calculated as the sum of all episodes in which the mothers were looking at, swiping on, or typing on the smartphone screen. The total amount of time spent in performing the smartphone task was calculated from the moment mothers first picked up the smartphone—or began using it without physically picking it up—until they completed their response to the last message. The scores of behaviors to maintain contact were calculated as percentage of time these behaviors were exhibited during the entire period of performing the smartphone task. The videos were coded by two trained coders, and the reliability was tested on 21.2% of the videos. The analyses showed an excellent agreement between the coders for the duration of smartphone use (ICC = 0.99), multitasking duration (ICC = 1.00) and task switching duration (ICC = 0.94).

### 2.5 Data analysis

Repeated measures ANOVAs and a MANOVA with the within-subjects factor phase (free play phase vs. interruption phase) and the between-subjects factor condition (order of phases) were conducted to test the hypotheses concerning differences between the two experimental phases. The between-subjects factor condition was included to control for potential order effects. The interaction effect (phase × condition) was examined to control if the effect of phase varied depending on the order in which the phases were presented.

To investigate the exploratory research questions relating to expected cross-sectional associations, correlation analyses were conducted. We assessed the assumptions for computing Pearson’s *r* and determined that the relations between the variables were not linear. Therefore, we calculated Kendall’s *τ* coefficient, which offers a more robust measure of association in the presence of nonlinearity (Field, 2013).

The data were screened for potential outliers by creating histograms, boxplots and scatterplots for dependent variables. Two cases with extremely high negative affect scores were identified. To assess their influence on the results of the analysis of variance, a bootstrapping analysis was conducted using R version 4.2.1 (R Core Team, 2024). Since the inclusion of these cases did not affect the significance of the results, we decided to retain these cases in the final sample.

All further analyses were performed with IBM SPSS Statistics, version 28.0.0.0 (IBM Corp, 2021).

## 3 Results

### 3.1 Comparison between the experimental phases

In the following, the results of the analyses on the comparisons of the free play and the interruption phase are reported; descriptive statistics and *F*, *p* and η_p_^2^ values are presented in Table 2.

**Table 2:** Descriptive statistics of the dependent variables and ANOVA results (*N* = 52).

Variable	M (SD)				ANOVA								
	Free play phase		Interruption phase		Phase			Condition			Phase × condition		
	Condition 1	Condition 2	Condition 1	Condition 2	*F*	*p*	η_p_^2^	*F*	*p*	η_p_^2^	*F*	*p*	η_p_^2^
Positive affect score	7.05 (11.49)	8.17 (11.02)	1.68 (3.73)	3.61 (6.50)	9.59	0.003	0.16	0.69	0.411	0.01	0.06	0.804	0.00
Negative affect score	9.70 (17.26)	27.24 (51.13)	22.92 (22.58)	15.87 (18.11)	0.03	0.860	0.00	0.612	0.438	0.01	5.58	0.022	0.10
Sensitivity	5.75 (1.24)	5.31 (1.28)	5.29 (1.03)	5.10 (1.01)	6.92	0.011	0.12	1.19	0.282	0.02	0.96	0.333	0.02
Structuring	5.96 (1.16)	5.29 (1.18)	5.12 (1.33)	4.87 (1.10)	19.86	<0.001	0.28	2.38	0.130	0.05	2.21	0.144	0.04
Nonintrusiveness	6.21 (1.06)	5.69 (1.37)	6.38 (0.90)	6.08 (1.16)	3.74	0.059	0.07	2.18	0.146	0.04	0.54	0.467	0.01
Nonhostility	6.87 (0.39)	6.46 (0.95)	6.56 (0.61)	6.52 (0.92)	2.36	0.131	0.05	1.32	0.255	0.03	5.05	0.029	0.09
Child responsiveness	5.52 (1.40)	5.00 (1.48)	5.02 (1.20)	5.15 (1.13)	1.23	0.273	0.02	0.35	0.559	0.01	4.38	0.041	0.08
Child involvement	4.00 (1.24)	3.75 (1.19)	3.52 (1.05)	3.48 (0.89)	6.21	0.016	0.11	0.29	0.590	0.01	0.49	0.485	0.01
Frequency of responses (%)	95.69 (7.27)	95.43 (9.62)	72.87 (23.04)	76.34 (22.96)	48.43	<0.001	0.49	0.18	0.671	0.00	0.38	0.539	0.01
Latency of responses ^a^ (s)	0.90 (0.59)	0.88 (0.77)	1.84 (1.38)	1.49 (0.90)	21.52	<0.001	0.31	0.73	0.397	0.02	0.96	0.333	0.02

a *n* = 50. Condition 1: order free play phase - interruption phase, Condition 2: order interruption phase - free play phase.

#### 3.1.1 Infant affect

Two 2 × 2 repeated measures ANOVAs were performed to examine the effect of the experimental phase on each the infants’ positive and negative affect score (hypothesis 1), controlling for the order of phases (condition) and the phase × condition interaction. For the infants’ positive affect score, there was neither a significant main effect of condition nor a significant phase × condition interaction. For the infants’ negative affect score, there was no main effect of condition; however a significant phase × condition interaction was found, suggesting that the main effect of phase depends on condition.

**Figure 1:**
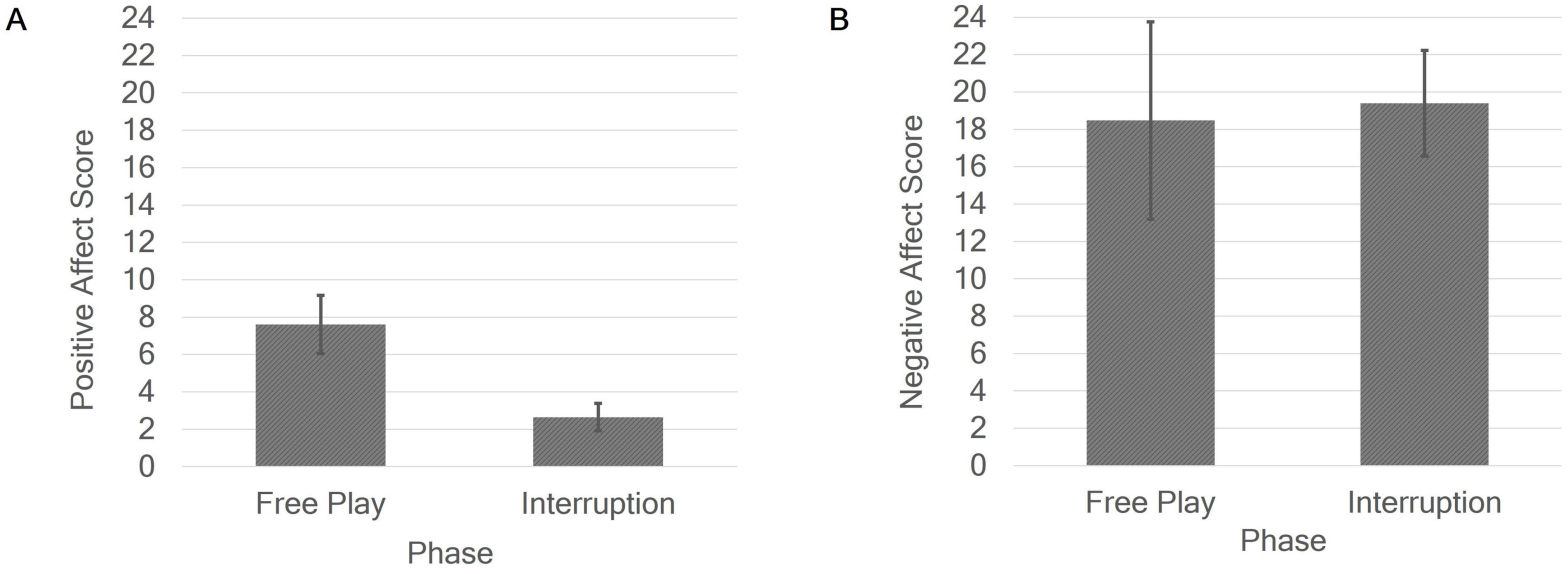
Main effects of phase on positive **(A)** and negative **(B)** affect scores. Error bars show standard error.

**Figure 2:**
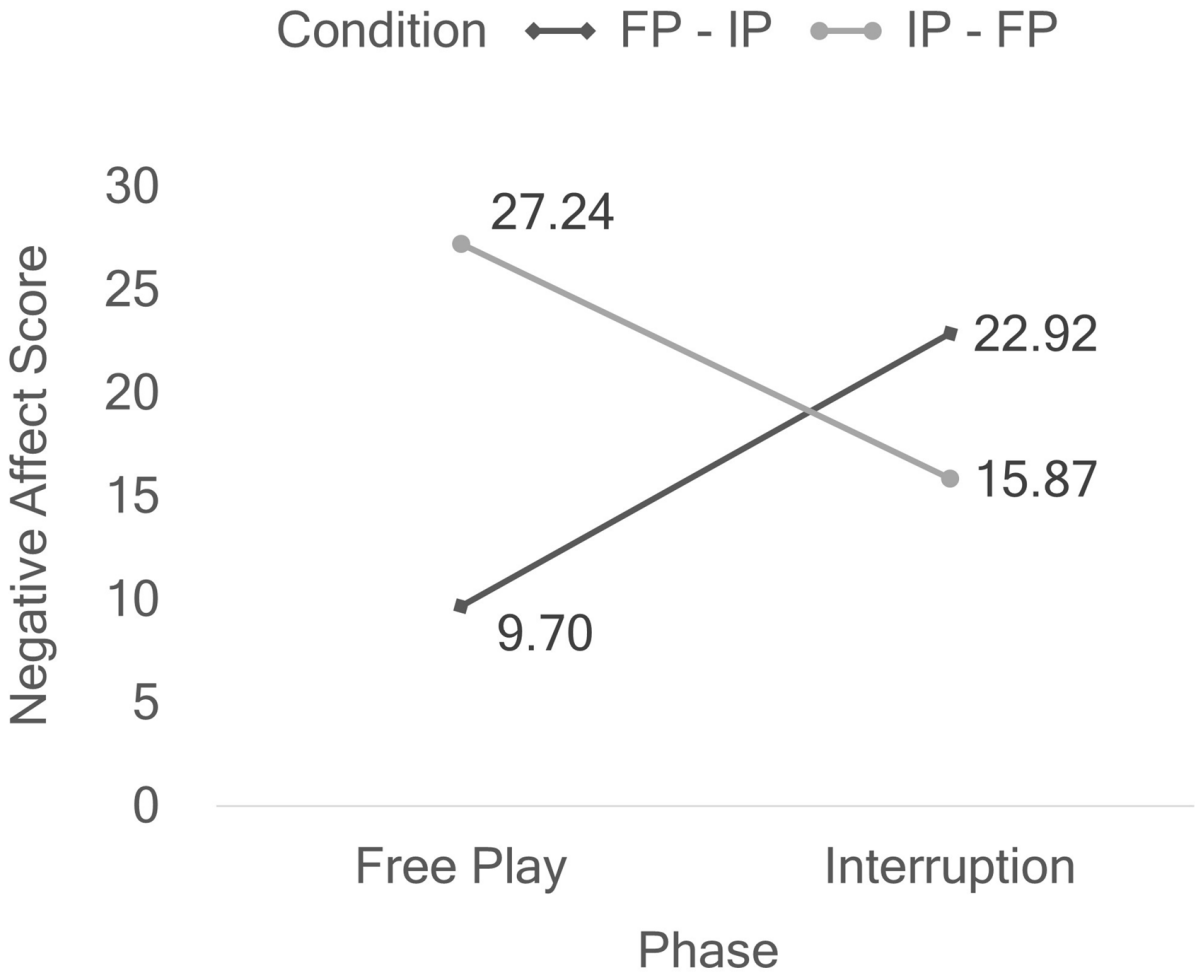
Interaction effect phase × condition on negative affect score. FP, free play phase, IP, interruption phase.

The effects of phase on infants’ positive and negative affect scores are shown in Figure 1. The first ANOVA revealed a significant effect of phase on the infants’ positive affect score: Infants expressed significantly less positive affect in the interruption phase (*M* = 2.64, *SD* = 5.33) than in the free play phase of the experiment (*M* = 7.61, *SD* = 11.16, η_p_^2^ = 0.16). The second ANOVA showed no significant effect of phase on the infants’ negative affect score (*p* = 0.860). However, this result should be interpreted in the context of the interaction effect phase × condition (Figure 2). To further explore this interaction effect on the infants’ negative affect score, we conducted paired-samples one-tailed t-tests separately for the two conditions. The analysis revealed a significant effect of phase on the infants’ negative affect score in condition 1, with a medium effect size, *t*(25) = −3.02, *p* = 0.003, *d* = −0.59. In contrast, no significant effect of phase was observed in condition 2, *t*(25) = 1.20, *p* = 0.120. Specifically, in condition 1, when the interruption phase followed the free play phase, the infants’ negative affect increased in the interruption phase (*M* = 22.92, *SD* = 22.58) compared to the free play phase (*M* = 9.70, *SD* = 17.26). However, in condition 2, when the interruption phase preceded the free play phase, the infants’ negative affect was relatively stable across both phases with interruption phase (*M* = 15.87, *SD* = 18.11) and free play phase (*M* = 27.24, *SD* = 51.13) showing comparable levels of negative affect.

#### 3.1.2 EA of the dyad

The mean scores regarding both phases ranged from *M* = 5.19 to *M* = 5.53 for sensitivity, which indicates a somewhat inconsistent to bland sensitivity, *M* = 4.99 to *M* = 5.63 for structuring, indicating a somewhat inconsistent to moderate level of structuring, *M* = 5.95 to *M* = 6.23 for nonintrusiveness, indicating a generally nonintrusive style of the interaction, *M* = 6.54 to *M* = 6.66 for nonhostility, indicating a generally nonhostile style of interaction, *M* = 5.09 to *M* = 5.26 for child responsiveness, indicating a somewhat complicated to moderate optimal responsiveness, and *M* = 3.50 to *M* = 3.88 for child involvement, indicating a somewhat not optimal to complicated involvement.

A 2 × 2 repeated measures MANOVA was performed to examine the effect of the experimental phase on the EA dimensions (hypothesis 2 and exploratory research question 1), controlling for the order of phases (condition) and the phase × condition interaction. There was neither a significant multivariate effect of condition (*F*(6, 45) = 0.89, *p* = 0.507), nor a significant interaction effect phase × condition (F(6, 45) = 1.36, *p* = 0.251). The analysis revealed a significant multivariate effect of phase on the combined dependent variables, *F*(6, 45) = 5.78, *p* < 0.001, with a large effect size (η_p_^2^ = 0.44).

**Figure 3:**
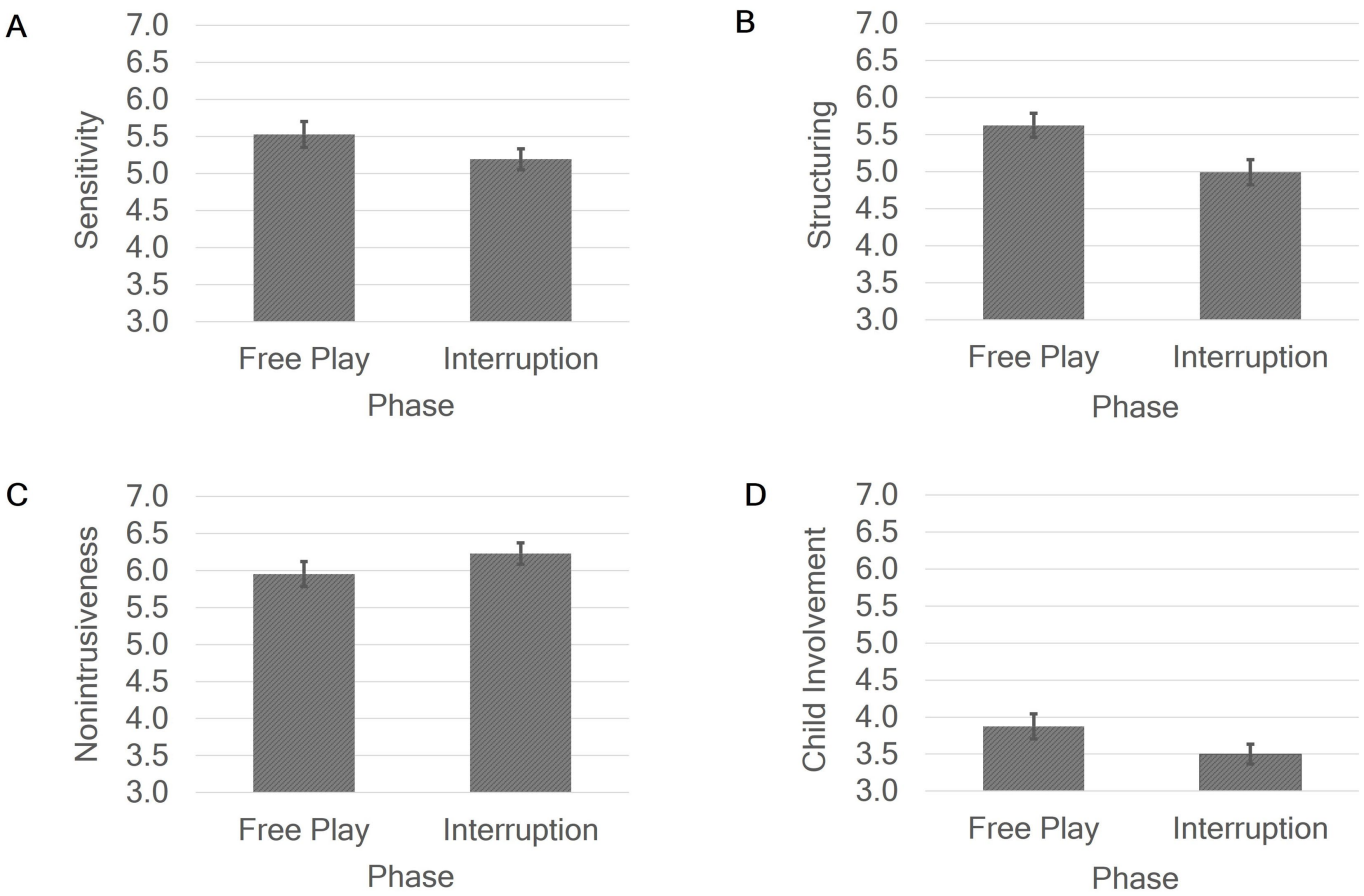
Main effects of phase on sensitivity **(A)**, structuring **(B)**, nonintrusiveness **(C)** and child involvement **(D)**. Error bars show standard error.

In the next step, separate ANOVAs were conducted for each EA dimension to assess the effect of phase, while controlling for condition and the phase × condition interaction. No effects of condition and no interaction effects phase × condition were found for the dimensions of sensitivity, structuring, nonintrusiveness and child involvement. The analyses revealed a significant main effect of phase on sensitivity (η_p_^2^ = 0.12), structuring (η_p_^2^ = 0.28), and child involvement (η_p_^2^ = 0.11; see Figure 3). Mothers expressed significantly less sensitivity in the interruption phase (*M* = 5.19, *SD* = 1.02) than in the free play phase of the experiment (*M* = 5.53, *SD* = 1.27). Furthermore, mothers showed significantly less optimal structuring in the interruption phase (*M* = 4.99, *SD* = 1.21) than in the free play phase (*M = 5.63, SD =* 1.21). In addition, children showed significantly less well involvement of their mothers in the interruption phase (*M* = 3.50, *SD* = 0.97) than in the free play phase (*M* = 3.88, *SD* = 1.21). Lastly, we found a marginally significant main effect of phase on maternal nonintrusiveness (*p* = 0.059, η_p_^2^ = 0.07; see Figure 3). Mothers expressed more nonintrusiveness in the interruption phase (*M* = 6.23, *SD* = 1.04), than in the free play phase of the experiment (*M* = 5.95, *SD* = 1.24).

For the other two EA dimensions, no main effects of condition were found but significant interaction effects of phase × condition emerged for nonhostility (η_p_^2^ = 0.09) and child responsiveness (η_p_^2^ = 0.08). However, since the multivariate interaction effect was not significant, we refrain from interpreting these results. No main effects of phase were observed for these two dimensions.

#### 3.1.3 Maternal responsiveness

The frequency of infants’ signals mothers responded to in the free play phase ranged from 60 to 100% and in the interruption phase from 0 to 100%. The range of the latency of responses in the free play phase was from 0.12 to 3.52 s, and in the interruption phase from 0.24 to 5.68 s.

**Figure 4:**
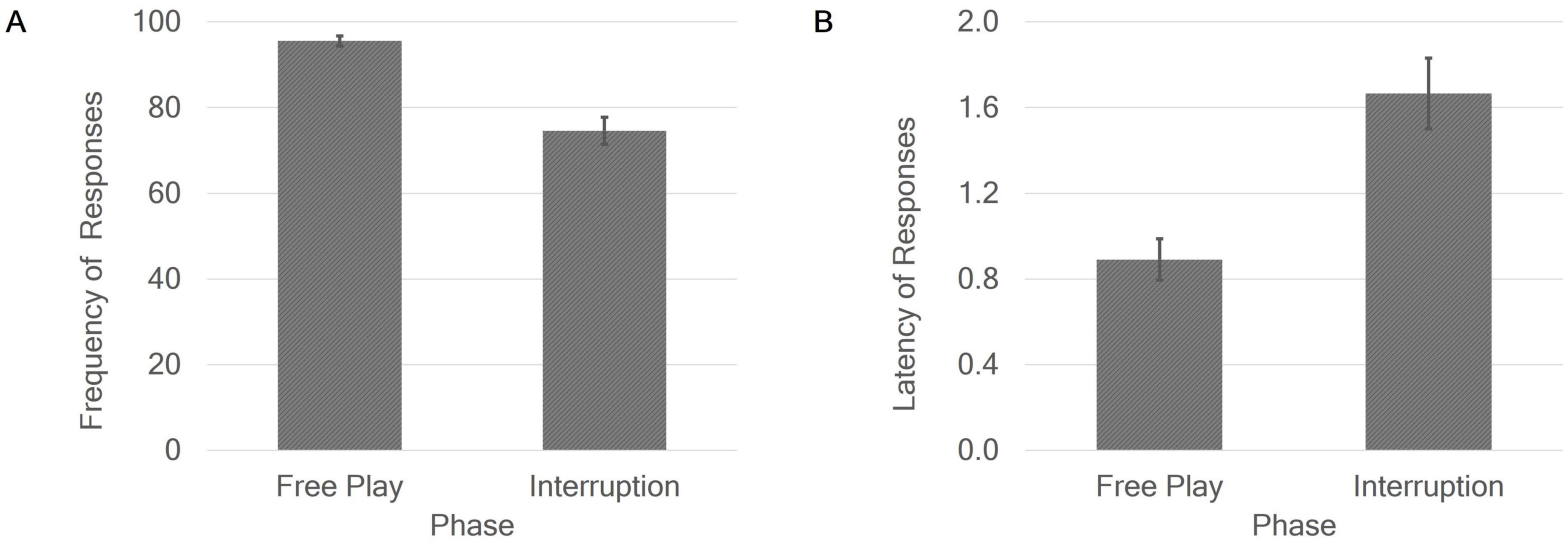
Main effects of phase on frequency of responses in % **(A)** and latency of responses in seconds **(B)**. Error bars show standard error.

Two 2 × 2 repeated measures ANOVAs were conducted to measure the effects of experimental phase on frequency of responses and latency of responses (hypothesis 3), controlling for the order of phases (condition) and the phase × condition interaction. There were neither significant main effects of condition nor significant interaction effects phase × condition on the frequency of responses or the latency of responses. The effects of phase on frequency of responses and latency of responses are shown in Figure 4. The first ANOVA showed a significant main effect of phase on the frequency of responses, with a large effect size (η_p_^2^ = 0.49): Mothers responded to less signals of their children in the interruption phase (74.6%) than in the free play phase of the experiment (95.6%). The second ANOVA revealed a significant main effect of phase on the latency of responses with a large effect size (η_p_^2^ = 0.31), whereby mothers responded significantly slower to the infants’ signals in the interruption phase (*M* = 1.67, *SD* = 1.17) than in the free play phase (*M* = 0.89, *SD* = 0.68).

### 3.2 Relations between maternal behavior and infant affect

On average, mothers spent 3.34 min (*SD* = 0.99) using the smartphone during the interruption phase. The average amount of time spent performing the smartphone task was 4.38 min (*SD* = 1.29 min). During the smartphone task, mothers spent an average of 30.5% of the time actively maintaining contact with their children (range from 0 to 92.9%); 15.5% involved active multitasking and 14.5% involved active task switching. The average percentage of time spent passively maintaining contact with children was 73.1% (range from 0 to 99.4%), whereby 59.6% was spent in passive multitasking, and 13.6% in passive task switching. Active and passive behaviors could be shown simultaneously.

Bivariate correlation analyses (Kendall’s *τ*) were conducted to examine the associations between the duration of smartphone use as well as maternal active and passive behaviors to maintain contact in the interruption phase and the infants’ positive and negative affect scores (research question 2). There were no significant correlations between the duration of smartphone use and the infants’ positive affect score (*τ*(50) = −0.08, *p* = 0.484), and between percentage of active and passive behaviors to maintain contact and the infants’ positive affect score (*τ*(50) = −0.03, *p* = 0.754; *τ*(50) = 0.01, *p* = 0.919, respectively). In addition, there was no correlation between the infants’ positive affect score and the percentage of active multitasking (*τ*(50) = −0.04, *p* = 0.699) or the percentage of active task switching (*τ*(50) = 0.00, *p* = 0.993), nor with the percentage of passive multitasking (*τ*(50) = 0.05, *p* = 0.679) or the percentage of passive task switching (*τ*(50) = 0.00, *p* = 0.978). However, there was a significant negative correlation between the duration of smartphone use and the infants’ negative affect score, *τ*(50) = −0.22, *p* = 0.026: A longer duration of smartphone use was associated with less infants’ negative affect. Furthermore, there was a significant positive correlation between the percentage of active behaviors to maintain contact and the infants’ negative affect score, *τ*(50) = 0.30, *p* = 0.003: More active behaviors to maintain contact were associated with more infants’ negative affect. Analyzing the separate scores, only active task switching correlated significantly with the infants’ negative affect score, *τ*(50) = 0.31, *p* = 0.001, whereas active multitasking did not significantly correlate with the infants’ negative affect score (*τ*(50) = 0.15, *p* = 0.127). There was no significant correlation between percentage of passive behaviors to maintain contact and the infants’ negative affect score, *τ*(50) = 0.06, *p* = 0.572. The passive task switching score correlated significantly with the infants’ negative affect score, *τ*(50) = 0.31, *p* = 0.002, whereas passive multitasking did not correlate with the infants’ negative affect score (*τ*(50) = −0.02, *p* = 0.855).

## 4 Discussion

The aim of this study was to investigate the impact of mothers’ smartphone use in presence of their children aged 5 to 6 months on children’s affect regulation and the quality of mother–child interactions as indicated by the EA of the dyad and maternal responsiveness. Additionally, we exploratively examined associations between mothers’ behaviors to maintain contact with children and children’s affective states. We hypothesized that infants would show less positive and more negative affect and that their mothers would display lower levels of sensitivity and responsiveness in the interruption phase compared to the free play phase of the experiment. This was predominantly supported by our results which showed significantly lower infants’ positive affect in the interruption phase compared to the free play phase, as well as significantly higher negative affect in the interruption phase, in case the interruption phase followed the free play phase. Moreover, as expected, mothers showed lower levels of sensitivity and responsiveness in the interruption phase than in the free play phase. In addition, we found mothers’ structuring of the interaction to be less optimal and children’s involvement of their mothers to be worse in the interruption phase compared to the free play phase. Lastly, we found associations between the duration of mothers’ smartphone use and active behaviors to maintain contact with their children and the children’s negative affect.

Regarding children’s affect regulation (hypothesis 1), our findings showed that infants expressed significantly less positive affect in the interruption phase compared to the free play phase. However, this result needs to be interpreted with caution as positive affect was generally low in this sample. In addition, there was a significant increase in infants’ negative affect in the interruption phase in case the interruption phase followed the free play phase. This is in line with previous findings on children’s affective response to their parents’ PDD use (Konrad et al., 2021a; Rozenblatt-Perkal et al., 2022). In contrast to previous findings (Konrad et al., 2021a; Rozenblatt-Perkal et al., 2022), infants in our study did not show a significant decrease in negative affect in the free play phase following the interruption phase. Repeated episodes of mothers’ smartphone use may have caused multiple disruptions to the interaction, leading to increased frustration in the infants that required time to recover. This could be interpreted as a carry-over effect—affective changes induced during the interruption phase that persisted into the free play phase. An alternative explanation would be that the infants may have become fatigued toward the end of the experiment. Further research is needed to draw definitive conclusions. Overall, our findings indicate that interruptions of the mother–child interaction due to the mothers’ smartphone use can negatively impact infants’ affect regulation, and that the effect might last beyond the interruption period. If such repeated episodes of mothers’ smartphone use occur often in everyday life, infants might spend a considerable amount of time trying to draw their mothers’ attention or self-regulate affect, at the cost of play and exploration (e.g., Tronick, 1989). This might in turn have negative consequences for the further development of the children’s capacity to self-regulate affect.

Concerning the quality of mother–infant interactions, as expected, mothers showed less sensitivity in the interruption phase than in the free play phase of the experiment (hypothesis 2). This indicates that repeated texting reduces maternal sensitivity during the usage period, or leads to an inconsistent sensitivity, presumably by reducing the clarity of perceptions of the infants’ behavioral and emotional signals, the quality of responses to them, and/or maternal warmth and consistency in the affective expression. This result adds to the findings of observational studies (Tharner et al., 2022; Wolfers et al., 2020) by shedding light on the causal relationships between maternal PDD use and reduced sensitivity. Moreover, mothers responded to significantly less children’s vocal signals in the interruption phase than in the free play phase of the experiment (hypothesis 3), which is consistent with the results of studies with toddlers (Konrad et al., 2021b; Lederer et al., 2022). In addition, they responded slower to the infants’ signals when using a smartphone in comparison to the free play situation. Infants are highly sensitive to the timing of interpersonal interactions (e.g., Jaffe et al., 2001). If adults’ reactions occur beyond the period mutual responses typically occur (within half to 3 sec after the signal; Beebe et al., 2010, Van Egeren et al., 2001), it might be difficult for infants to perceive it as related to their behavior (Millar and Watson, 1979). Although in our study the mean latency of mothers’ responses in the interruption phase did not exceed 3 sec, some individual response times fell outside this range. This suggests that in some cases, the mothers’ reaction to their children’s vocal signals occurred too late, so that the children could no longer understand their reaction as such. Considering the importance of maternal sensitivity and responsiveness during the first year of life for children’s social–emotional development, repeated maternal PDD use in presence of their children might have unfavorable consequences.

In addition, our results indicate that further aspects of EA of the mother–infant dyad are also affected by maternal smartphone use (research question 1). Mothers showed less optimal structuring in the interruption phase compared to the free play phase, suggesting that repeated texting impedes mothers’ ability to provide adequate scaffolding to their children. These findings are in line with the results of Corkin et al. (2021), showing that a higher frequency of audible notifications on parents’ PDD in everyday life was associated with lower parental scaffolding observed in a free play. Apart from that, we found a marginally significant effect of the experimental phase on maternal nonintrusiveness. Mothers tended to be less intrusive during the interruption phase than during the free play phase. Similarly to the effect on maternal structuring, PDD use seems to limit mothers’ ability to actively engage in the interaction, in this case leading to a more positive outcome—less interference in the children’s activities. However, as this effect did not reach statistical significance and as nonintrusiveness was generally high in this sample, more evidence is needed to substantiate such an interpretation. Maternal smartphone use also affected children’s contributions to the interaction. Generally, children’s level of involving their mothers in the interaction was low in this sample due to their young age and limited verbal and motor capacities. However, the children showed even more non-optimal involvement in the interruption phase compared to the free play phase. Hence, maternal PDD use seems to lead to more negatively involving behaviors (such as protest) or uninvolving behaviors.

Finally, we found that shorter maternal smartphone use was associated with more infants’ negative affect, suggesting that mothers react to their infants’ distress by reducing smartphone use. Mothers actively tried to maintain contact with their children during approximately one third of the time spent performing the smartphone task, whereas they did so passively during almost three quarters of the time. They did so partly through multitasking and partly through making breaks in usage to attend to their children. A higher amount of maternal active (but not passive) behaviors to maintain contact was associated with more negative affect expressed by the infants (research question 2). A closer examination of the associations between mothers’ behaviors to maintain contact and their children’s negative affect revealed that mothers’ task switching behaviors, but not their multitasking behaviors were associated with infants’ negative affect: More mothers’ active as well as passive task switching was associated with more infants’ negative affect. While the correlational analysis does not allow us to determine the direction of influence, we propose two possible explanations for this finding. Mothers may have extended breaks to attend to their children in response to their distress. Alternatively, task switching could have disrupted the children. Specifically, repeated interruptions in the flow of interaction due to mothers shifting focus might have disturbed the continuity of the infants’ experience, possibly causing confusion and frustration.

Overall, the results suggest that although mothers seem to adapt smartphone use based on their children’s affective response, the disruption to the interaction due to mothers’ repeated smartphone use negatively impacts their children’s affect regulation and impedes the quality of the mother–child interactions.

This study has several strengths. We used a controlled setting, which enabled us to draw inferences about causal relations between maternal smartphone use and children’s affect regulation and the quality of the mother–child interactions. In addition, the experiment was designed to correspond to everyday situations as much as possible, by allowing mothers to coordinate smartphone use and the interaction with their children, so that a high ecological validity was achieved. Moreover, both global (EAS) and event-based assessments (responsiveness) were employed to reduce the impacts of singular methodological approaches on study outcomes. Finally, we systematically investigated mothers’ behaviors to maintain contact with their children and thereby addressed an existing gap of research.

However, several limitations of the study should be considered. Most of the sample consisted of dyads with a high SES, limiting the generalizability of the findings to the general population. Apart from this, our study focused exclusively on the effects of text messaging. The increasingly widespread use of voice messaging was not examined but may be less disruptive than text messaging. Recording voice messages, similar to a phone call, could enable parents to maintain eye contact with their children (Radesky et al., 2014). In addition, although the experiment was designed to maximize ecological validity, the generalizability of the results to real-world settings remains limited. For example, the social desirability bias might have impacted mothers’ behavior. In real-life situations, mothers may be even more likely to overlook their children’s signals while responding to a text message. Alternatively, mothers could postpone their response to a more appropriate moment, e.g., when their children are interacting with another family member. Furthermore, our findings do not allow us to disentangle the specific effects of smartphone use from the broader impact of repeated interruptions. Including a non-digital distraction condition in future studies could offer valuable insight into the differential effects of digital versus non-digital parental distraction. In addition, to simulate a situation that closely resembles everyday life, we opted for relatively open instructions regarding the mothers’ task during the interruption phase. As a result, the findings revealed considerable variability in mothers’ behavior during the experimental task, as reflected by the wide range of behaviors to maintain contact with children during smartphone use, as well as in the response latencies to their children’s signals. Future studies could incorporate additional conditions with standardized instructions to systematically vary maternal behavior. Lastly, our research design does not permit inferences about the directionality of the relations between maternal behaviors to maintain contact with their children and the children’s negative affect. Further research is needed to elucidate these associations.

The results of this study suggest that mothers’ repeated smartphone use for text messaging in presence of their children aged 5 to 6 months could negatively impact children’s affective states during and beyond the period of usage. Furthermore, our findings show that mothers’ smartphone use decreases the quality of mother–infant interactions as indicated by the EA of the dyad and by maternal responsiveness. Moreover, we found evidence that mothers attempt to coordinate smartphone use with the interaction with their children: While performing the smartphone task, mothers spent a significant amount of time interacting with children, both simultaneously and by making breaks to attend to the children. We also found some evidence that mothers adjust their smartphone use in response to their children’s affective reactions—by reducing the duration of use and by engaging more actively with their children while using the smartphone.

However, further research is needed to examine how dividing attention between children and a PDD over an extended period affects the quality of parent–child interactions and children’s affect regulation. Furthermore, it is important to determine the extent of parental PDD use and the specific patterns of use that may have a disruptive effect. Finally, the impact of parental PDD use in the presence of children on the development of children’s capacity for affective self-regulation remains to be explored.

## Data Availability

The raw data supporting the conclusions of this article will be made available by the authors, without undue reservation.

## References

[ref1] Ainsworth M. D. S. Blehar M. C. Waters E. Wall S. (1978). Patterns of attachment: A psychological study of the strange situation. Hillsdale, NJ: Erlbaum Associates.

[ref2] Alvarez Gutierrez S. Ventura A. K. (2021). Associations between maternal technology use, perceptions of infant temperament, and indicators of mother-to-infant attachment quality. Early Hum. Dev. 154:105305. doi: 10.1016/j.earlhumdev.2021.105305, PMID: 33508559

[ref3] Beebe B. Jaffe J. Markese S. Buck K. Chen H. Cohen P.. (2010). The origins of 12-month attachment: a microanalysis of 4-month mother-infant interaction. Attach Hum. Dev. 12, 3–141. doi: 10.1080/14616730903338985, PMID: 20390524 PMC3763737

[ref5] Bergmann S. von Klitzing K. Keitel-Korndörfer A. Wendt V. Grube M. Herpertz S.. (2016). Emotional availability, understanding emotions, and recognition of facial emotions in obese mothers with young children. J. Psychosom. Res. 80, 44–52. doi: 10.1016/j.jpsychores.2015.11.005, PMID: 26721547

[ref6] Biringen Z. (2008). The emotional availability (EA) scales. 4th Edn. Boulder, CO: International Center for Excellence in Emotional Availability.

[ref7] Biringen Z. Derscheid D. Vliegen N. Closson L. Easterbrooks M. A. (2014). Emotional availability (EA): theoretical background, empirical research using the EA scales, and clinical applications. Dev. Rev. 34, 114–167. doi: 10.1016/j.dr.2014.01.002

[ref8] Bornstein M. H. Tamis-LeMonda C. S. (1997). Maternal responsiveness and infant mental abilities: specific predictive relations. Infant Behav. Dev. 20, 283–296. doi: 10.1016/S0163-6383(97)90001-1

[ref9] Bornstein M. H. Tamis-LeMonda C. S. Tal J. Ludemann P. Toda S. Rahn C. W.. (1992). Maternal responsiveness to infants in three societies: the United States, France, and Japan. Child Dev. 63, 808–821. doi: 10.2307/1131235, PMID: 1505242

[ref10] Bosquet Enlow M. B. King L. Schreier H. M. Howard J. M. Rosenfield D. Ritz T.. (2014). Maternal sensitivity and infant autonomic and endocrine stress responses. Early Hum. Dev. 90, 377–385. doi: 10.1016/j.earlhumdev.2014.04.007, PMID: 24794304 PMC4065189

[ref11] Bosquet Enlow M. B. Kitts R. L. Blood E. Bizarro A. Hofmeister M. Wright R. J. (2011). Maternal posttraumatic stress symptoms and infant emotional reactivity and emotion regulation. Infant Behav. Dev. 34, 487–503. doi: 10.1016/j.infbeh.2011.07.007, PMID: 21862136 PMC3180882

[ref12] Braune-Krickau K. Schneebeli L. Pehlke-Milde J. Gemperle M. Koch R. von Wyl A. (2021). Smartphones in the nursery: parental smartphone use and parental sensitivity and responsiveness within parent-child interaction in early childhood (0-5 years): a scoping review. Infant Ment. Health J. 42, 161–175. doi: 10.1002/imhj.21908, PMID: 33452702 PMC8048888

[ref13] Cabañero L. Hervás R. González I. Fontecha J. Mondéjar T. Bravo J. (2020). Characterisation of mobile-device tasks by their associated cognitive load through EEG data processing. Futur. Gener. Comput. Syst. 113, 380–390. doi: 10.1016/j.future.2020.07.013

[ref14] Calkins S. D. (2004). “Early attachment processes and the development of emotional self-regulation” in Handbook of self-regulation: Research, theory, and applications. eds. Baumeister R. F. Vohs K. D. (New York: The Guilford Press), 325–369.

[ref15] Cassidy J. (1994). Emotion regulation: influences of attachment relationships. Monogr. Soc. Res. Child Dev. 59, 228–249. doi: 10.1111/j.1540-5834.1994.tb01287.x 7984163

[ref16] Chamam S. Forcella A. Musio N. Quinodoz F. Dimitrova N. (2024). Effects of digital and non-digital parental distraction on parent-child interaction and communication. Front Child Adolesc Psychiatry 3:1330331. doi: 10.3389/frcha.2024.1330331, PMID: 39839318 PMC11748799

[ref17] Cole P. M. Michel M. K. Teti L. O. D. (1994). The development of emotion regulation and dysregulation: a clinical perspective. Monogr. Soc. Res. Child Dev. 59, 73–100. doi: 10.2307/1166139, PMID: 7984169

[ref18] Corkin M. T. Henderson A. M. E. Peterson E. R. Kennedy-Costantini S. Sharplin H. S. Morrison S. (2021). Associations between technoference, quality of parent-infant interactions, and infants’ vocabulary development. Infant Behav. Dev. 64:101611. doi: 10.1016/j.infbeh.2021.101611, PMID: 34303915

[ref19] Davis M. I. Delfosse C. M. Ventura A. K. (2022). Infant age moderates associations between infant temperament and maternal technology use during infant feeding and care. Int. J. Environ. Res. Public Health 19:858. doi: 10.3390/ijerph191912858, PMID: 36232158 PMC9565936

[ref20] Faul F. Erdfelder E. Lang A.-G. Buchner A. (2007). G*power 3: a flexible statistical power analysis program for the social, behavioral, and biomedical sciences. Behav. Res. Methods 39, 175–191. doi: 10.3758/BF03193146, PMID: 17695343

[ref21] Feldman R. Singer M. Zagoory O. (2010). Touch attenuates infants’ physiological reactivity to stress. Dev. Sci. 13, 271–278. doi: 10.1111/j.1467-7687.2009.00890.x, PMID: 20136923

[ref22] Field A. P. (2013). Discovering statistics using IBM SPSS statistics. 4th Edn. London: SAGE Publications.

[ref23] Fitz N. Kushlev K. Jagannathan R. Lewis T. Paliwal D. Ariely D. (2019). Batching smartphone notifications can improve well-being. Comput. Human Behav. 101, 84–94. doi: 10.1016/j.chb.2019.07.016

[ref24] Fonagy P. Gergely G. Jurist E. Target M. (2005). Affect regulation, mentalization, and the development of the self. New York: Other Press.

[ref25] Gergen K. J. (2002). “The challenge of absent presence” in Perpetual contact: Mobile communication, private talk, public performance. eds. Katz J. E. Aakhus M. (Cambridge: Cambridge University Press), 227–241.

[ref26] Gianino A. Tronick E. Z. (1988). “The mutual regulation model: the infant’s self and interactive regulation and coping and defensive capacities” in Stress and coping across development. eds. Field T. M. McCabe P. M. Schneiderman N. (Hillsdale, NJ: Erlbaum), 47–68.

[ref27] Goldsmith D. F. Rogoff B. (1997). Mothers’ and toddlers’ coordinated joint focus of attention: variations with maternal dysphoric symptoms. Dev. Psychol. 33, 113–119. doi: 10.1037/0012-1649.33.1.113, PMID: 9050396

[ref28] Gusella J. L. Muir D. Tronick E. Z. (1988). The effect of manipulating maternal behavior during an interaction on three-and six-month-olds’ affect and attention. Child Dev. 59, 1111–1124. doi: 10.2307/1130278, PMID: 3168619

[ref29] IBM Corp (2021). IBM SPSS statistics for windows, version 28.0. Armonk, NY: IBM Corp.

[ref30] Inoue C. Hashimoto Y. Nakatani Y. Ohira M. (2022). Smartphone use during breastfeeding and its impact on mother-infant interaction and maternal responsiveness: within-subject design. Nurs. Health Sci. 24, 224–235. doi: 10.1111/nhs.12918, PMID: 34941018

[ref31] Jaffe J. Beebe B. Feldstein S. Crown C. L. Jasnow M. D. Rochat P.. (2001). Rhythms of dialogue in infancy: coordinated timing in development. Monogr. Soc. Res. Child Dev. 66, 1–149. 11428150

[ref380] Keenan K. (2000). Emotion dysregulation as a risk factor for child psychopathology. Clin. Psychol. Sci. Prac. 7, 418–434. doi: 10.1093/clipsy.7.4.418

[ref33] Konrad C. Berger-Hanke M. Hassel G. Barr R. (2021a). Does texting interrupt imitation learning in 19-month-old infants? Infant Behav. Dev. 62:101513. doi: 10.1016/j.infbeh.2020.101513, PMID: 33338985

[ref34] Konrad C. Hillmann M. Rispler J. Niehaus L. Neuhoff L. Barr R. (2021b). Quality of mother-child interaction before, during, and after smartphone use. Front. Psychol. 12:616656. doi: 10.3389/fpsyg.2021.616656, PMID: 33854461 PMC8039320

[ref35] Lampert T. Hoebel J. Kuntz B. Müters S. Kroll L. E. (2018). Messung des sozioökonomischen Status und des subjektiven sozialen Status in KiGGS Welle 2. J. Health Monit. 3, 114–133. doi: 10.17886/RKI-GBE-2018-0162018

[ref36] Lederer Y. Artzi H. Borodkin K. (2022). The effects of maternal smartphone use on mother-child interaction. Child Dev. 93, 556–570. doi: 10.1111/cdev.13715, PMID: 34807453

[ref38] Lyons-Ruth K. (1999). The two-person unconscious: intersubjective dialogue, enactive relational representation, and the emergence of new forms of relational organization. Psychoanal. Inq. 19, 576–617. doi: 10.1080/07351699909534267

[ref39] Mangold P. (2018). “Discover the invisible through tool-supported scientific observation (translation of: das Unsichtbare entdecken durch werkzeuggestützte wissenschaftliche Beobachtung)” in Mindful evolution. Conference proceedings. eds. Böttger H. Jensen K. Jensen T. (Bad Heilbrunn: Klinkhardt).

[ref40] McDaniel B. T. Coyne S. M. (2016). “Technoference”: the interference of technology in couple relationships and implications for women’s personal and relational well-being. Psychol. Pop. Media Cult. 5, 85–98. doi: 10.1037/ppm0000065

[ref41] Mesman J. van Ijzendoorn M. H. Bakermans-Kranenburg M. J. (2009). The many faces of the still-face paradigm: a review and meta-analysis. Dev. Rev. 29, 120–162. doi: 10.1016/j.dr.2009.02.001

[ref42] Mikić A. Klein A. M. (2022). Smartphone-Nutzung in Gegenwart von Babys und Kleinkindern: Ein systematisches Review. Prax. Kinderpsychol. Kinderpsychiatr. 71, 305–326. doi: 10.13109/prkk.2022.71.4.305 35673787

[ref43] Millar W. S. Watson J. S. (1979). The effect of delayed feedback on infant learning reexamined. Child Dev. 50, 747–751. doi: 10.2307/1128941, PMID: 498851

[ref44] Myruski S. Gulyayeva O. Birk S. Perez-Edgar K. Buss K. A. Dennis-Tiwary T. A. (2018). Digital disruption? Maternal mobile device use is related to infant social-emotional functioning. Dev. Sci. 21:e12610. doi: 10.1111/desc.12610, PMID: 28944600 PMC5866735

[ref45] Oulasvirta A. Rattenbury T. Ma L. Raita E. (2011). Habits make smartphone use more pervasive. Pers. Ubiquit. Comput. 16, 105–114. doi: 10.1007/s00779-011-0412-2

[ref46] R Core Team (2024). *R*: A language and environment for statistical computing. Vienna, Austria: R Foundation for Statistical Computing.

[ref47] Radesky J. S. Kistin C. J. Zuckerman B. Nitzberg K. Gross J. Kaplan-Sanoff M.. (2014). Patterns of mobile device use by caregivers and children during meals in fast food restaurants. Pediatrics 133, e843–e849. doi: 10.1542/peds.2013-3703, PMID: 24616357

[ref48] Rozenblatt-Perkal Y. Davidovitch M. Gueron-Sela N. (2022). Infants' physiological and behavioral reactivity to maternal mobile phone use – an experimental study. Comput. Human Behav. 127:107038. doi: 10.1016/j.chb.2021.107038

[ref49] Salvucci D. D. Taatgen N. A. Borst J. P. (2009) Toward a unified theory of the multitasking continuum: from concurrent performance to task switching, interruption, and resumption, in: CHI ‘09: Proceedings of the SIGCHI Conference on Human Factors in Computing Systems (Boston, MA: ACM Press), 1819–1828.

[ref50] Sameroff A. J. (2014). “A dialectic integration of development for the study of psychopathology” in Handbook of developmental psychopathology. eds. Lewis M. Rudolph K. D. (New York: Springer New York), 25–43.

[ref51] Saunders H. Biringen Z. Benton J. Closson L. Herndon E. Prosser J. L. (2017). Emotional availability and emotional availability zones (EA-Z): from assessment to intervention and universal prevention. Perspect. Infant Ment. Health 25, 12–16.

[ref52] Saunders H. Kraus A. Barone L. Biringen Z. (2015). Emotional availability: theory, research, and intervention. Front. Psychol. 6:1069. doi: 10.3389/fpsyg.2015.01069, PMID: 26283996 PMC4516809

[ref53] Sroufe L. A. (1996). Emotional development: The organization of emotional life in the early years. New York: Cambridge University Press.

[ref54] Stack D. M. LePage D. E. (1996). Infants’ sensitivity to manipulations of maternal touch during face-to-face interactions. Soc. Dev. 5, 41–55. doi: 10.1111/j.1467-9507.1996.tb00071.x

[ref55] Stack D. M. Muir D. W. (1992). Adult tactile stimulation during face-to-face interactions modulates five-month-olds’ affect and attention. Child Dev. 63, 1509–1525. doi: 10.2307/1131572, PMID: 1446566

[ref56] Stockdale L. A. Porter C. L. Coyne S. M. Essig L. W. Booth M. Keenan-Kroff S.. (2020). Infants’ response to a mobile phone modified still-face paradigm: links to maternal behaviors and beliefs regarding technoference. Infancy 25, 571–592. doi: 10.1111/infa.12342, PMID: 32857440

[ref57] Thai S. Page-Gould E. (2018). Experiencesampler: an open-source scaffold for building smartphone apps for experience sampling. Psychol. Methods 23, 729–739. doi: 10.1037/met0000151, PMID: 28616998

[ref58] Tharner A. Mortensen A. H. Holmsgaard E. M. Vaever M. S. (2022). Mothers’ smartphone use and mother-infant interactive behavior in the postpartum period. Pediatr. Res. 91, 8–11. doi: 10.1038/s41390-021-01451-4, PMID: 33731805

[ref59] Thompson R. A. (1994). Emotion regulation: a theme in search of definition. Monogr. Soc. Res. Child Dev. 59, 25–52. doi: 10.1111/j.1540-5834.1994.tb01276.x, PMID: 7984164

[ref60] Tronick E. (1989). Emotions and emotional communication in infants. Am. Psychol. 44, 112–119. doi: 10.1037/0003-066X.44.2.112, PMID: 2653124

[ref61] Tronick E. Als H. Adamson L. Wise S. Brazelton T. B. (1978). The infant’s response to entrapment between contradictory messages in face-to-face interaction. J. Am. Acad. Child Psychiatry 17, 1–13. doi: 10.1016/S0002-7138(09)62273-1, PMID: 632477

[ref62] van der Schuur W. A. Baumgartner S. E. Sumter S. R. Valkenburg P. M. (2015). The consequences of media multitasking for youth: a review. Comput. Human Behav. 53, 204–215. doi: 10.1016/j.chb.2015.06.035

[ref63] Van Egeren L. A. Barratt M. S. Roach M. A. (2001). Mother–infant responsiveness: timing, mutual regulation, and interactional context. Dev. Psychol. 37, 684–697. doi: 10.1037/0012-1649.37.5.684, PMID: 11552763

[ref64] Ventura A. K. Levy J. Sheeper S. (2019). Maternal digital media use during infant feeding and the quality of feeding interactions. Appetite 143:104415. doi: 10.1016/j.appet.2019.104415, PMID: 31445993

[ref65] Wade-Bohleber L. M. Braune-Krickau K. Schneebeli L. Gemperle M. Haechler R. Pehlke-Milde J.. (2024). Smartphone use during the perinatal period: findings from a longitudinal study with first-time parents. Comput. Human Behav. 154:127. doi: 10.1016/j.chb.2023.108127

[ref66] Wolfers L. N. Kitzmann S. Sauer S. Sommer N. (2020). Phone use while parenting: an observational study to assess the association of maternal sensitivity and smartphone use in a playground setting. Comput. Hum. Behav. 102, 31–38. doi: 10.1016/j.chb.2019.08.013

