# Supplementary material for: “Just a second, mommy’s here”: the impact of mothers’ smartphone use on children’s affect regulation and the quality of mother–child interactions
Source: Front Psychol. 2025 Jul 9;16:1596219. doi: 10.3389/fpsyg.2025.1596219 (PMC12283988; doi:10.3389/fpsyg.2025.1596219)
Supplement: Supplementary file 1 [file Table_1.docx]

**Supplementary Material**

**Supplementary Table 1.** Maintaining Contact Scale.

| Maternal Behavior | Descriptor |
| --- | --- |
| Monitoring | |
| Gaze | Looks at the child (and shows no other active behaviors to maintain contact) |
| Active maintaining contact | |
| Facial communication | Uses facial expressions (e.g., smiles, raises eyebrows) while looking at the child (and shows no other active behaviors to maintain contact) |
| Interaction with a hand | Uses hand movements to interact with the child (e.g., moves her hand over the child in a playful way, or moves fingers up and down) |
| Interaction with a toy | Manipulates a toy within the child’s sight (e.g., rolls, presses or shakes a toy, or hands a toy to the child) |
| Touch | Touches the child (including playful touching and stroking) |
| Kissing | Kisses the child |
| Talking | Speaks to the child, or comments on what she is doing |
| Singing | Makes musical sounds |
| Laughter | Laughs (in a communicative manner, not for herself) |
| Vocalization | Makes other communicative vocal sounds (e.g., makes soothing sounds, throws a kiss, whistles, mumbles) |
| Drawing the child near | Brings the child onto her lap, or positions the child to lean against her, or lifts the child into her arms |
| Calming movements | Rocks or sways the child |
| Repositioning | Adjusts the child’s position |
| Maternal Behavior | Descriptor |
| Passive maintaining contact | |
| Holding a hand up | Holds her hand up within the child’s sight |
| Holding a toy | Holds a toy up within the child’s sight |
| Sustained touch | Holds the child’s hand or another part of the child’s body, or the child holds the mother’s hand or another part of her body |
| Keeping the child near | The child stays on the mother’s lap, leaning against the mother or in her arms |
| Other | |
| Non-communicative  behaviors | Starts an action but does not complete it (e.g., reaches for a toy but pulls her hand back), or performs an action out of the child’s sight (e.g., manipulates or holds a toy that the child cannot see) |
